# Supplementary material for: Chromothripsis during telomere crisis is independent of NHEJ, and consistent with a replicative origin
Source: Genome Res. 2019 May;29(5):737–49. doi: 10.1101/gr.240705.118 (PMC6499312; doi:10.1101/gr.240705.118)
Supplement: Supplemental Material [file supp_gr.240705.118_Supplemental_file_1.zip › contigs/annotated_contigs/DB73/contig.2.DB73_length_525_mean_cov_9.24761904762.docx]

**DB73_length_525_mean_cov_9.24761904762**

```
GTCTCTAACCAACTGGTTATTTCTCCTTTTCTGCCCAAGACTAATGCCCTTCCAGATGAGGATCTTCACCTCAGTCATCACATCCTCTC
 >chr16:71981077-71981297 - E=9e-118 p=1e-02
TCCCAGTGCTTCATCTCTCCCCTGCATTTTATTTAATTTTATTTTTTTGAGACGGAGTTTCACTCTTGTTGTCAAGGCTGGAGTGCAAT

GATGTGATCTCAGCTCACTACAACCTCCACCTCCTGGGTT|CA|GAGGAGAGGATTGTGGAAACTACAATTCAAGATGAGATTTGGGTG
                                         >chr16:71989299-71989606 - E=4e-170
GGGATACAGCTAAGCCCTATTAGGTGTAGTGGTGTGCACCTGCACACCACTGAGGTGGGAGGATCACTTGAGCCAGGGGGATGGAGGTT

GCAATGAGCTGAGATCTCACCACTGCACTCCACTCCAACCTGGGTGACGCAGCAAGATCCTGTCTCAAAAACAAAAAACTAAATGATAT

TCTGTTATGTGTGTGTGTTTATATACACCCCTTTTTTTGTTTGTTTTTTAGATGGAGTCTCTCTGTAGCCCAGGCTGGAGTG
```
